# Morphology, genetic characterization and phylogeny of *Moniliformis tupaia* n. sp. (Acanthocephala: Moniliformidae) from the northern tree shrew *Tupaia belangeri chinensis* Anderson (Mammalia: Scandentia)

**DOI:** 10.1017/S0031182024000271

**Published:** 2024-04

**Authors:** Hui-Xia Chen, Zhi-Jun Yu, Jun Ma, Cui-Hong Zhao, Fu-Qiong Cao, Liang Li

**Affiliations:** 1 Hebei Collaborative Innovation Center for Eco-Environment, Hebei Key Laboratory of Animal Physiology, Biochemistry and Molecular Biology, College of Life Sciences, Hebei Normal University, 050024 Shijiazhuang, Hebei Province, P.R. China; 2 Ecological Postdoctoral Research Mobile Station, Hebei Normal University, 050024 Shijiazhuang, Hebei Province, P.R. China; 3 Hebei Research Center of the Basic Discipline Cell Biology, Ministry of Education Key Laboratory of Molecular and Cellular Biology, 050024 Shijiazhuang, Hebei Province, P.R. China; 4 Key Laboratory of Veterinary Public Health of Yunnan Province, College of Veterinary Medicine, Yunnan Agricultural University, Kunming, Yunnan Province 650201, P.R. China

**Keywords:** Acanthocephala, *Moniliformis*, morphology, phylogeny, Tupaiidae

## Abstract

A new species of *Moniliformis*, *M. tupaia* n. sp. is described using integrated morphological methods (light and scanning electron microscopy) and molecular techniques (sequencing and analysing the nuclear 18S, ITS, 28S regions and mitochondrial *cox*1 and *cox*2 genes), based on specimens collected from the intestine of the northern tree shrew *Tupaia belangeri chinensis* Anderson (Scandentia: Tupaiidae) in China. Phylogenetic analyses show that *M. tupaia* n. sp. is a sister to *M. moniliformis* in the genus *Moniliformis*, and also challenge the systematic status of *Nephridiacanthus major*. *Moniliformis tupaia* n. sp. represents the third *Moniliformis* species reported from China.

## Graphical abstract

**Figure:**
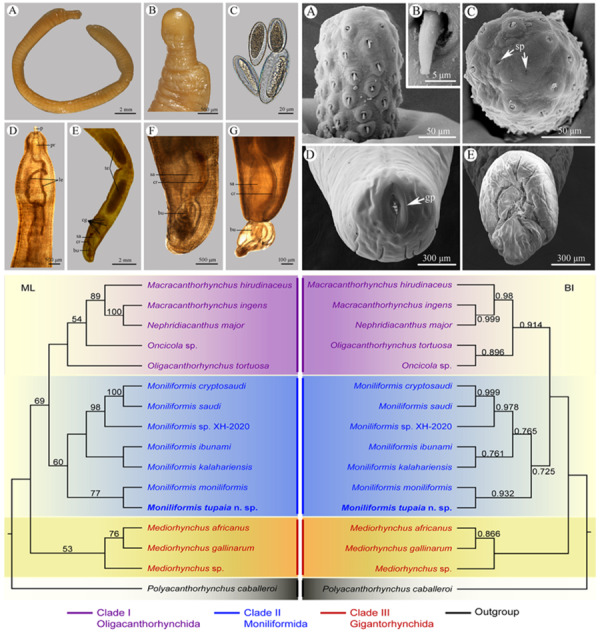

## Introduction

The northern tree shrew *Tupaia belangeri chinensis* Anderson (Mammalia: Scandentia: Tupaiidae) is a novel ideal animal model for human disease, due to its small size, easy breeding, rapid reproduction and close genetic relationship to primates (Xu *et al*., 2012, 2013; Xiao *et al*., 2017; Tang *et al*., 2018; Wang *et al*., 2021). *Tupaia belangeri chinensis* is omnivorous, eating fruits, seeds, insects and small vertebrates, which is mainly distributed in southwest China (including Yunnan and Sichuan Provinces) (Xiang and Yang, 2014) and can act as the intermediate and definitive host for some helminth parasites and protozoa (Brack *et al*., 1987; Tian *et al*., 1989; Xiang *et al*., 2010; Xiang and Yang, 2014). However, our present knowledge of the species composition of the acanthocephalans of the northern tree shrew is very limited. To date, only *Prosthenorchis* sp. (Archiacanthocephala: Oligacanthorhynchidae) has been reported from *T. belangeri chinensis* (Tian *et al*., 1989).

In the present study, some acanthocephalan specimens were collected from *T. belangeri chinensis* in China. In order to accurately identify these acanthocephalan specimens to species level, the detailed morphology of these specimens was studied using light and scanning electron microscopy. Moreover, the nuclear small subunit ribosomal DNA (18S), internal transcribed spacer (ITS) and large subunit ribosomal DNA (28S), and mitochondrial cytochrome c oxidase subunit 1 (*cox*1) and subunit 2 (*cox*2) genes were sequenced and analysed. Phylogenetic analyses were also performed based on the 18S + *cox*1 sequence data using maximum likelihood (ML) and Bayesian inference (BI) methods, to clarify the phylogenetic relationships between this species and its congeners.

## Materials and methods

### Morphological observation

Acanthocephalans were isolated from the intestine of the northern tree shrew *T. belangeri chinensis* in Kunming, Yunnan Province, China. Specimens were washed and kept in cold water for several hours until the proboscis everted, and then stored in 80% ethanol until studied. For light microscopical studies, specimens were made in impermanent mount slide and cleared in lactophenol. Photomicrographs were recorded using a Nikon® digital camera coupled to a Nikon® optical microscopy. For scanning electron microscopy (s.e.m.), specimens were post-fixed in 1% OsO4, dehydrated *via* an ethanol series and acetone, and then critical point dried. The specimens were coated with gold and examined using a Hitachi S-4800 scanning electron microscope at an accelerating voltage of 20 Kv. Measurements (range, followed by the mean in parentheses) are given in micrometres unless otherwise stated.

### Molecular procedures

Genomic DNA from the mid-body of one male and one female was extracted using a Column Genomic DNA Isolation Kit (Shanghai Sangon, China) according to the manufacturer's instructions. The partial 18S region was amplified by polymerase chain reaction (PCR) using the forward primer (5′-AGATTAAGCCATGCATGCGTAAG-3′) and the reverse primer (5′-TGATCCTTCTGCAGGTTCACCTAC-3′) (Garey *et al*., 1996). The partial 28S region was amplified by PCR using 4 overlapping PCR fragments of 700–800 bp. Primers for 28S amplicon 1 were forward 5′-CAAGTACCGTGAGGGAAAGTTGC-3′ and reverse 5′-CAGCTATCCTGAGGGAAAC-3′; amplicon 2, forward 5′-ACCCGAAAGATGGTGAACTATG-3′ and reverse 5′-CTTCTCCAAC(T/G)TCAGTCTTCAA-3′; amplicon 3, forward 5′-CTAAGGAGTGTGTAACAACTCACC-3′ and reverse 5′-AATGACGAGGCATTTGGCTACCTT-3′; amplicon 4, forward 5′-GATCCGTAACTTCGGGAAAAGGAT-3′ and reverse 5′-CTTCGCAATGATAGGAAGAGCC-3′ (García-Varela and Nadler, 2005). The partial ITS region was amplified by PCR using the forward primer (5'-GTCGTAACAAGGTTTCCGTA-3') and the reverse primer (5'-TATGCTTAAATTCAGCGGGT-3') (Král'ová-Hromadová *et al*., 2003). The partial *cox*1 region was amplified by PCR using the forward primer (5'-GGTCAACAAATCATAAAGATATTGG-3') and the reverse primer (5'-TAAACTTCAGGGTGACCAAAAAATCA-3') (Gómez *et al*., 2002). The partial *cox*2 region was amplified by PCR using the forward primer (5'-AATGTTTGATGGGTTTAGAG-3') and the reverse primer (5'-AACACTGACCATATATAACC-3') (designed by the present study). The cycling conditions were as described previously (Li *et al*., 2019). PCR products were checked on GoldView-stained 1.5% agarose gels and purified with Column PCR Product Purification Kit (Shanghai Sangon, China). Sequencing for each amplification product was carried out from both directions. Sequences were aligned using ClustalW2 and adjusted manually. The DNA sequences obtained herein were compared (using the algorithm BLASTn) with that available in the National Center for Biotechnology Information (NCBI) database (http://www.ncbi.nlm.nih.gov).

### Phylogenetic analyses

Phylogenetic analyses were performed based on the 18S + *cox*1 sequence data using maximum likelihood (ML) inference with IQ-TREE and Bayesian inference (BI) with Mrbayes 3.2 (Ronquist *et al*., 2012; Nguyen *et al*., 2015), respectively. *Polyacanthorhynchus caballeroi* Diaz-Ungria & Rodrigo, 1960 (Polyacanthocephala: Polyacanthorhynchida) was treated as the out-group. The in-group included 15 species of the class Archiacanthocephala representing 6 different genera belonging to 3 orders Gigantorhynchida, Moniliformida and Oligacanthorhynchida. The detailed information of acanthocephalan species included in the present phylogenetic analyses is provided in Table 1.

**Table 1.** Species of Archiacanthocephala with their detailed information of genetic data included in the phylogenetic analyses

Species	Host	Locality	GenBank ID for 18S region	GenBank ID for *cox*1 region	References
**Ingroup**					
Order Gigantorhynchida					
Family Gigantorhynchidae					
*Mediorhynchus africanus*	*Numida meliagris* (Aves: Galliformes)	Africa	KC261353	KC261351	Amin *et al*., 2013
*Mediorhynchus gallinarum*	*Gallus gallus* (Aves: Galliformes)	Indonesia	KC261354	KC261352	Amin *et al*., 2013
*Mediorhynchus* sp.	*Cassidix mexicanus* (Aves: Passeriformes)	N/A	AF064816	AF416996	García-Varela *et al*., 2000, 2002
Order Moniliformida					
Family Moniliformidae					
*Moniliformis cryptosaudi*	*Hemiechinus auratus* (Mammalia: Erinaceomorpha)	Iraq	MH401043	MH401041	Amin *et al*., 2019
*Moniliformis ibunami*	*Peromyscus hylocetes* (Mammalia: Rodentia)	Mexico	MW136271	MW115576	Lynggaard *et al*., 2021
*Moniliformis kalahariensis*	*Atelerix frontalis* (Mammalia: Erinaceomorpha)	South Africa	MH401042	MH401040	Amin *et al*., 2019
*Moniliformis moniliformis*	*Rattus norvegicus* (Mammalia: Rodentia); N/A	England; N/A	Z19562	AF416998	Telford and Holland, 1993; García-Varela *et al*., 2002
*Moniliformis saudi*	*Paraechinus aethiopicus* (Mammalia: Erinaceomorpha)	Saudi Arabia	KU206782	KU206783	Amin *et al*., 2016
*Moniliformis* sp. XH-2020	*Eospalax fontanierii baileyi* (Mammalia: Rodentia)	China	OM388438	OK415026	Dai *et al*., 2022
*Moniliformis tupaia* n. sp.	*Tupaia belangeri* (Mammalia: Scandentia)	China	PP002170	OR997666	This study
Order Oligacanthorhynchida					
Family Oligacanthorhynchidae					
*Macracanthorhynchus hirudinaceus*	*Sus scrofa leucomystax* (Mammalia: Artiodactyla); *S. scrofa meridionalis* (Mammalia: Artiodactyla)	Japan; Italy	LC350000	MZ683370	Kamimura *et al*., 2018; Dessì *et al*., 2022
*Macracanthorhynchus ingens*	N/A; *Procyon lotor* (Mammalia: Carnivora)	N/A; USA	AF001844	KT881244	Near *et al*., 1998; Richardson *et al*., 2016
*Nephridiacanthus major*	*Hemiechinus auratus* (Mammalia: Erinaceomorpha)	Iran	MN612079	MN612080	Amin *et al*., 2020
*Oligacanthorhynchus tortuosa*	*Didelphis virginiana* (Mammalia: Didelphimorphia)	N/A; Mexico	AF064817	KM659378	García-Varela *et al*., 2000; López-Caballero *et al*., 2015
*Oncicola* sp.	*Nasua narica* (Mammalia: Carnivora); N/A	N/A	AF064818	AF417000	García-Varela *et al*., 2000, 2002
Outgroup					
Class Polyacanthocephala					
Order Polyacanthorhynchida					
Family Polyacanthorhynchidae					
*Polyacanthorhynchus caballeroi*	*Caiman yacare* (Reptilia: Crocodylia)	Bolivia	AF388660	DQ089724	García-Varela *et al*., 2002; García-Varela and Nadler, 2006

We used a built-in function in IQTREE to select a best-fitting substitution model for the sequences according to the Bayesian information criterion (Posada and Crandall, 2001). The GTR + F + I + G4 model was identified as optimal nucleotide substitution model. Reliabilities for ML tree were tested using 1000 bootstrap replications and BI tree was tested using 10 million generations. In the ML tree, bootstrap support (BS) values ⩾90 were considered as fully supported; whereas BS values ⩾70 and <90 were considered as generally supported. In the BI tree, Bayesian posterior probabilities (BPP) ⩾0.90 were considered as fully supported, whereas BPP values ⩾0.70 and <0.90 were considered as generally supported.

## Results

### Description of *Moniliformis tupaia* n. sp. (×*Figs 1–3*)

#### General

**Figure 1.**
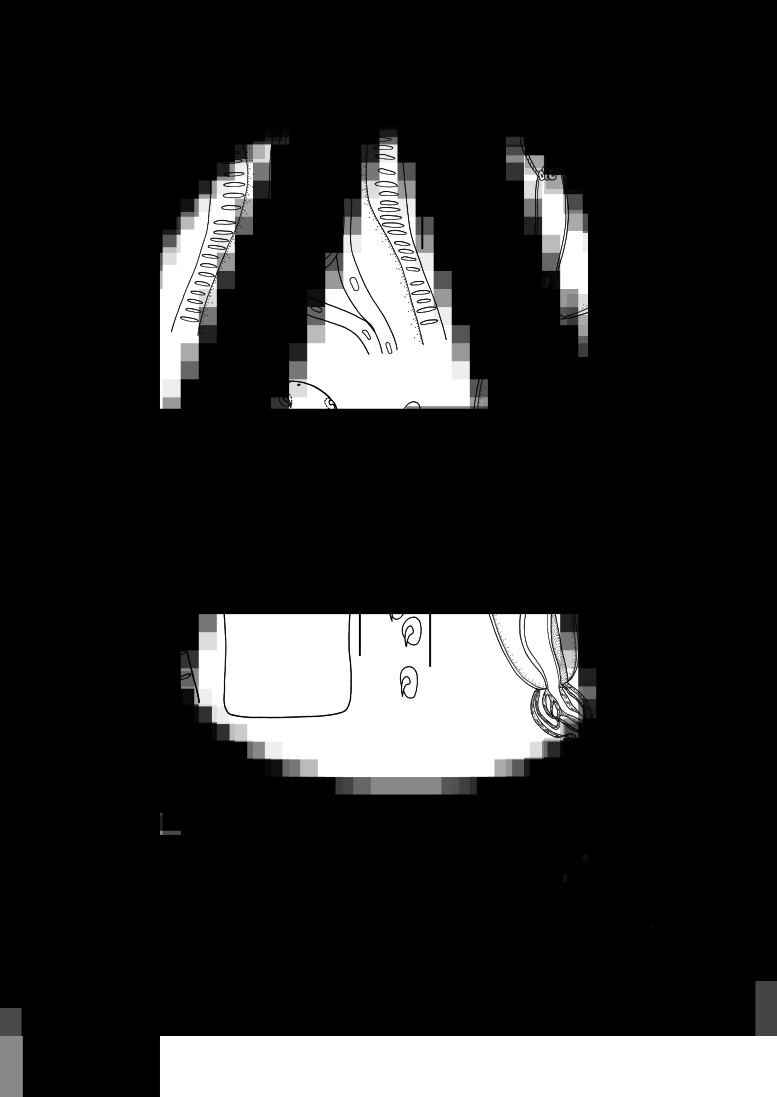
*Moniliformis tupaia* n. sp. collected from *Tupaia belangeri chinensis* (Scandentia: Tupaiidae) in China. (A) anterior part of male. (B) anterior end of male. (C) posterior end of female. (D) posterior part of male. (E) proboscis. (F) longitudinal row of hooks. (G) posterior end of male. (H) egg. *Scale bars*: A, D, G = 1000 *μ*m; B, C = 500 *μ*m; E, F = 100 *μ*m; H = 30 *μ*m.

**Figure 2.**
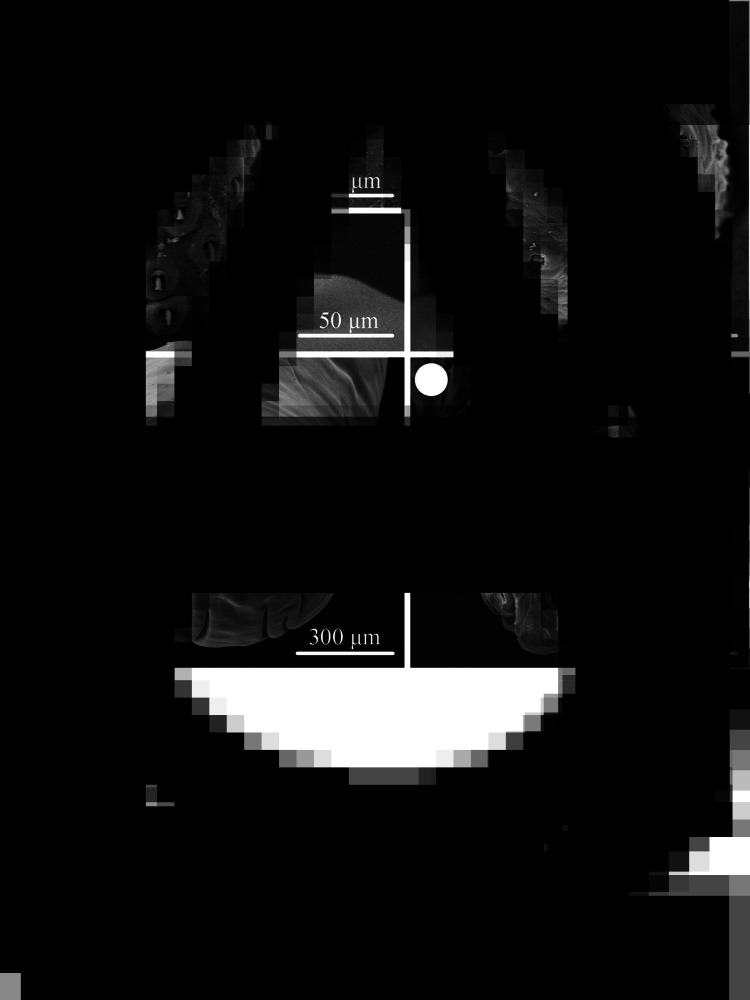
Scanning electron micrographs of *Moniliformis tupaia* n. sp. collected from *Tupaia belangeri chinensis* (Scandentia: Tupaiidae) in China. (A) Proboscis of male, lateral view. (B) Magnified image of proboscis hook. (C) Proboscis of male (sensory pores arrowed), apical view. (D) Posterior end of female (gonopore arrowed). (E) Copulatory bursa. *Abbreviations*: sp, sensory pores; gp, gonopore.

**Figure 3.**
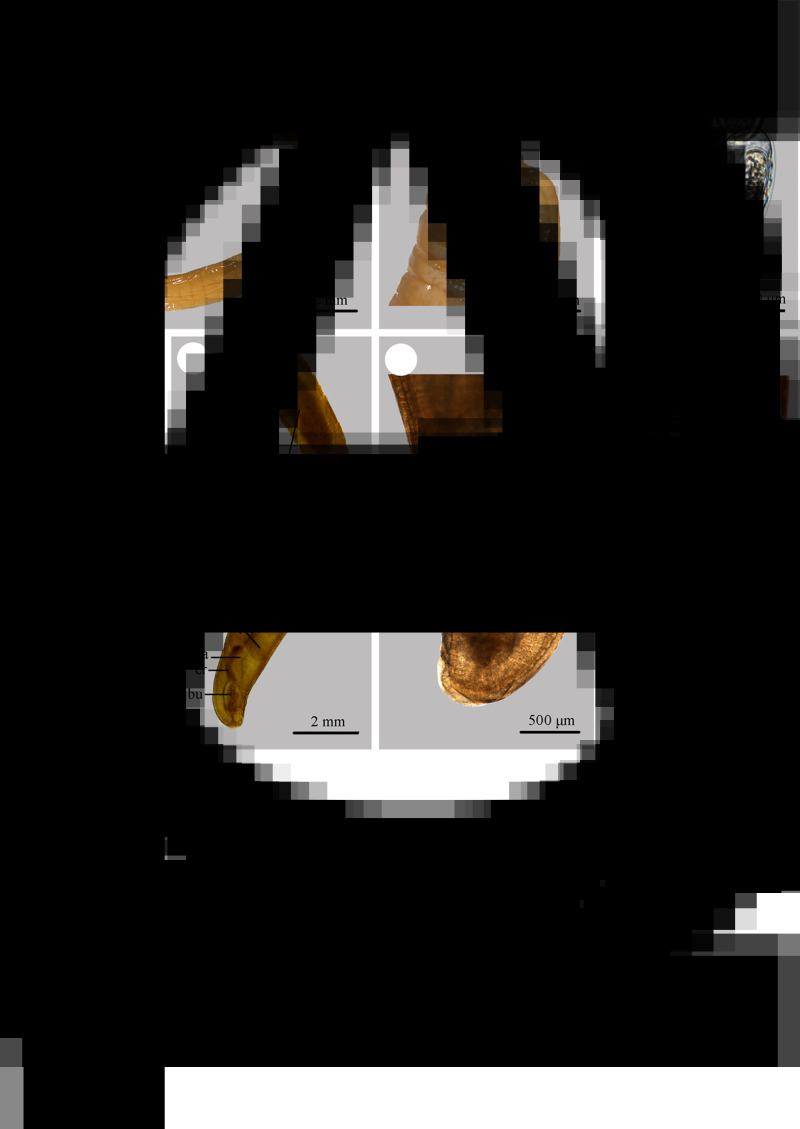
Photomicrographs of *Moniliformis tupaia* n. sp. collected from *Tupaia belangeri chinensis* (Scandentia: Tupaiidae) in China. (A) Mature female. (B) Tail of female. (C) Eggs. (D) Anterior part of male. (E) Posterior part of male. (F) Posterior end of male (copulatory bursa not everted). (G) Posterior end of male (copulatory bursa evaginabled). *Abbreviations*: bu, bursa; cg, cement glands; cr, cement reservoir; le, lemnisci; p, proboscis; pr, proboscis receptacle; sa, saeftigen's pouch; te, testis.

Medium-sized worms with small proboscis (Figs 1A; 3A, D). Trunk aspinose, nearly cylindrical and slender, showing pseudosegmentation characteristic of the genus *Moniliformis* (Fig. 3A). Anterior trunk tapering to gourd-shaped. Proboscis small compared to the trunk, cylindrical, with two apical sensory pores and 14 spiral longitudinal rows of 7–8 hooks each (Figs 1A, B, E; 2A, C; 3B). Proboscis hooks small, with simple roots (Figs 1E, F; 2A–C). Proboscis receptacle double-walled, cerebral ganglion at base of proboscis receptacle (Figs 1A, B; 3D). Neck short. Lemnisci very long, unequal, distinctly longer than proboscis receptacle (Figs 1A; 3D). Gonopore terminal in both sexes (Figs 1C, D; 2D).

#### **Male** [based on 5 mature specimens]

Trunk 34.0–47.5 (40.0) mm long, maximum width 1.83–2.07 (1.93) mm. Proboscis 366–439 (395) long, 146–171 (162) wide. Proboscis hooks similar in shape, 27–31 (30), 28–31 (29), 26–29 (28), 24–27 (26), 21–25 (24), 18–23 (21), 18–22 (20), 17–22 (19) in length from anteriorly to posteriorly. Neck 49–100 (68) long, 180–244 (204) wide. Proboscis receptacle 854–1195 (1000) long, 341–390 (368) wide. Shorter lemniscus 5.00–6.10 (5.72) mm long, longer lemniscus 7.68–9.32 (8.61) mm long. Testes two, oval, nearly equal in size; anterior testis 2.44–3.49 (3.05) mm long, 732–1024 (888) wide; posterior testis 2.44–3.54 (2.99) mm long, 585–1000 (849) wide (Fig. 1D). Cement glands eight, ovoid, clustered together; a short distance from posterior testis, 854–1829 (1256) long, 659–854 (761) wide (Figs 1D, G; 3E). Saefftigen's pouch 927–1512 (1317) long, 293–463 (378) wide. Copulatory bursa evaginabled or not everted, 780–1171 (971) long, 366–854 (644) wide (Figs 1G, 2E; 3F, G). Gonopore nearly terminal (Fig. 1D, G).

**Female** [Based on 1 mature specimen]. Trunk 41.0 mm long, maximum width 2.15 mm. Proboscis 390 long, 171 wide. Proboscis hooks similar in shape, 28–33 (31), 29–33 (32), 27–31 (29), 26–29 (27), 25–29 (27), 23–26 (24), 22–26 (24), 20–25 (22) in length from anteriorly to posteriorly. Neck 73 long, 195 wide. Proboscis receptacle 927 long, 439 wide. Shorter lemniscus 4.63 mm long, longer lemniscus 8.54 mm long. Uterine bell 350 long, 300 wide. Uterus 680 long, vagina 270 long (Fig. 1C). Eggs ellipsoid, 58–68 (65) × 24–32 (30) in size (*n* = 20) (Figs 1H; 3C). Gonopore nearly terminal (Figs 1C, 2D).

##### ***Type-host*:** Northern tree shrew *Tupaia belangeri chinensis* Anderson (Scandentia: Tupaiidae). ***Type-locality*:** Kunming, Yunnan Province, China. ***Site in host*:** Intestine. ***Type specimens*:** Holotype, male (HBNU-A-M20231201CL); allotype, female (HBNU-A-M20231202CL); paratypes: 4 males (HBNU-A-M20231203CL); deposited in the College of Life Sciences, Hebei Normal University, Hebei Province, China. ***Etymology:*** The species name refers to the generic name of the type host.

### Molecular characterization

#### Partial 18s region

Two 18S sequences of *M. tupaia* n. sp. obtained herein are both 1188 bp in length, with no nucleotide divergence detected. In the genus *Moniliformis*, there are 6 species with their 18S sequences available in GenBank, including *M. cryptosaudi* Amin, Heckmann, Sharifdini and Albayati, 2019 (MH401043), *M. ibunami* Lynggaard, García-Prieto, Guzmán-Cornejo and García-Varela, 2021 (MW136271, MW136272), *M. kalahariensis* Meyer, 1931 (MH401042), *M. moniliformis* (Bremser, 1811) (HQ536017, Z19562), *M. saudi* Amin, Heckmann, Mohammed and Evans, 2016 (KU206782) and *Moniliformis* sp. XH-2020 (OM388438). Pairwise comparison of the 18S sequences of *M. tupaia* n. sp. obtained herein with that of other *Moniliformis* species showed no nucleotide divergence (*Moniliformis* sp. XH-2020) to 0.66% (*M. ibunami*) nucleotide divergence. The 18S sequences of *M. tupaia* n. sp. obtained herein were deposited in GenBank database (http://www.ncbi.nlm.nih.gov) (under accession numbers PP002170, PP002171).

#### Partial 28s region

Two 28S sequences of *M. tupaia* n. sp. obtained herein are both 2692 bp in length, with no nucleotide divergence detected. In the genus *Moniliformis*, there are *M. ibunami* (MW136276, MW136277) and *M. moniliformis* (AY829086) with 28S sequences available in GenBank. Pairwise comparison of the 28S sequences of *M. tupaia* n. sp. obtained herein with that of other *Moniliformis* species showed 1.49% (*M. ibunami*) to 2.04% (*M. moniliformis*) nucleotide divergence. The 28S sequences of *M. tupaia* n. sp. obtained herein were deposited in GenBank database (http://www.ncbi.nlm.nih.gov) (under accession numbers PP002172, PP002173).

#### Partial ITS region

Two ITS sequences of *M. tupaia* n. sp. obtained herein are both 671 bp in length, with no nucleotide divergence detected. In the genus *Moniliformis*, only *M. moniliformis* (AF416415) has an ITS sequence available in GenBank. Pairwise comparison of the ITS sequences of *M. tupaia* n. sp. obtained herein with that of *M. moniliformis* showed 17.2% nucleotide divergence. The ITS sequences of *M. tupaia* n. sp. obtained herein were deposited in GenBank database (http://www.ncbi.nlm.nih.gov) (under accession numbers PP002174, PP002175).

#### Partial *cox*1 region

Two *cox*1 sequences of *M. tupaia* n. sp. obtained herein are both 658 bp in length, with no nucleotide divergence detected. In the genus *Moniliformis*, 7 species have their *cox*1 sequences available in GenBank, namely *M. cryptosaudi* (MH401041), *M. ibunami* (MW115575, MW115576), *M. kalahariensis* (MH401040), *M. moniliformis* (AF416998), *M. necromysi* Gomes, Costa, Gentile, Vilela and Maldonado, 2020 (MT803593), *M. saudi* (KU206783, OQ078755) and *Moniliformis* sp. XH-2020 (OK415026). Pairwise comparison of the *cox*1 sequences of *M. tupaia* n. sp. obtained herein with that of other *Moniliformis* species showed 24.9% (*M. ibunami*) to 27.3% (*M. moniliformis*) nucleotide divergence. The *cox*1 sequences of *M. tupaia* n. sp. obtained herein were deposited in GenBank database (http://www.ncbi.nlm.nih.gov) (under accession numbers OR997666, OR997667).

#### Partial *cox*2 region

Two *cox*2 sequences of *M. tupaia* n. sp. obtained herein are both 514 bp in length, with no nucleotide divergence detected. In the genus *Moniliformis*, only *Moniliformis* sp. XH-2020 (OK415026) has a *cox*2 sequence available in GenBank. Pairwise comparison of the *cox*2 sequences of *M. tupaia* n. sp. obtained herein with that of *Moniliformis* sp. XH-2020 showed 23.6% nucleotide divergence. The *cox*2 sequences of *M. tupaia* n. sp. obtained herein were deposited in GenBank database (http://www.ncbi.nlm.nih.gov) (under accession numbers PP002935, PP002936).

### Phylogenetic analyses

**Figure 4.**
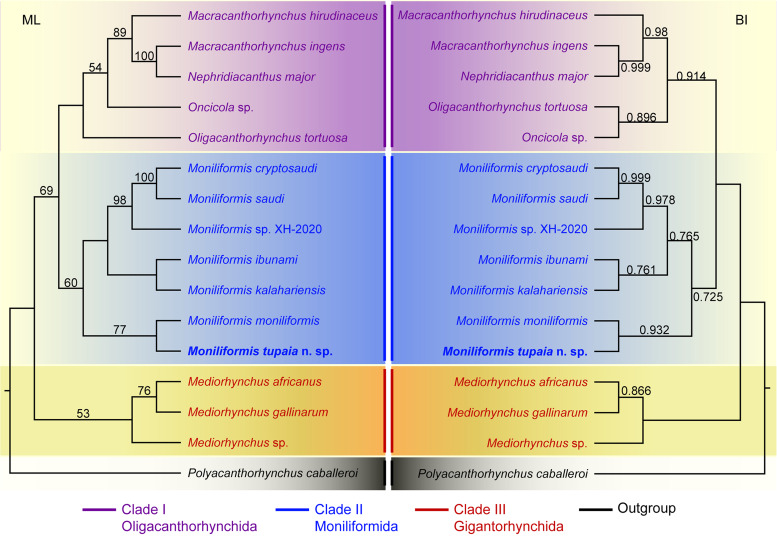
Maximum likelihood (ML) and Bayesian inference (BI) based on the 18S + *cox*1 sequence data showing the phylogenetic relationships of representatives of Archiacanthocephala. *Polyacanthorhynchus caballeroi* (Polyacanthocephala: Polyacanthorhynchidae) was chose as the outgroup. Bootstrap support (BS) values ⩾50 in ML tree and Bayesian posterior probabilities (BPP) ⩾ 0.70 in BI tree are shown.

Phylogenetic trees of the class Archiacanthocephala constructed from the 18S + *cox*1 sequence data using ML and BI methods have almost identical topology (Fig. 4). The representatives of Archiacanthocephala were divided into three major clades. Clade I included species of *Macracanthorhynchus*, *Nephridiacanthus*, *Oligacanthorhynchus* and *Oncicola*, representing the order Oligacanthorhynchida. Among them, the phylogenetic results showed *N. major* (Bremser, 1811) clustered together with *M. ingens* (Von Linstow, 1879). Clade I contained species of *Moniliformis*, representing the order Moniliformida. Clade III included species of *Mediorhynchus*, representing the order Gigantorhynchida. In the genus *Moniliformis*, *M. tupaia* n. sp. showed sister relationship with *M. moniliformis.*

## Discussion

The present specimens collected from the northern tree shrew *T. belangeri chinensis* belong to the genus *Moniliformis* (Moniliformida: Moniliformidae), due to the pseudosegmented trunk, the very small cylindrical proboscis, the double-walled proboscis receptacle, the very long lemnisci and the presence of 8 spherical cement glands (Travassos, 1917; Van Cleave, 1923, 1953; Southwell and Macfie, 1925; Yamaguti, 1963; Schmidt, 1972; Amin, 1987). The genus *Moniliformis* currently comprises 19 species mainly parasitic in mammals (Amin, 2013; Amin *et al*., 2016, 2019; Martins *et al*., 2017; Gomes *et al*., 2020; Lynggaard *et al*., 2021). Among them, only *M. moniliformis* and *Moniliformis* sp. XH-2020 have been reported in China (Chen, 1933; Chandler, 1941; Dai *et al*., 2022).

The proboscis of the new species has 14 spiral longitudinal rows of 7–8 simple rooted hooks each, which is similar to the proboscis of following species *M. acomysi* Ward and Nelson, 1967, *M. cryptosaudi*, *M. moniliformis*, *M. saudi* and *M. siciliensis* Meyer, 1932. *Moniliformis tupaia* n. sp. can be easily distinguished from *M. acomysi* by its much longer proboscis and lemnisci (proboscis 0.37–0.44 mm and lemnisci 5.00–9.32 mm long in the male of new species *vs* proboscis 0.19–0.36 mm and lemnisci 2.73–4.42 mm long in the male of *M. acomysi*). *Moniliformis tupaia* n. sp. differs from *M. cryptosaudi* and *M. saudi* by having larger cement glands (854–1829 long in the new species *vs* 312–811 long in the latter two species). Moreover, *M. cryptosaudi* and *M. saudi* are both parasitic in hedgehogs (Erinaceomorpha: Erinaceidae) in Saudi Arabia and Iraq, but the new species parasitizes the northern tree shrew *T. belangeri chinensis* in China. Furthermore, molecular analysis revealed strong genetic divergence (25.9‒26.9% difference in nucleotides in the *cox*1 region) between the new species and *M. cryptosaudi* and *M. saudi*. *Moniliformis siciliensis* is a poorly known acanthocephalan species only reported from the garden dormouse *Eliomys quercinus* Linnaeus (Mammalia: Rodentia) in the Italian island of Sicily (Meyer, 1932; Petrochenko, 1958). The new species differs from *M. siciliensis* in having shorter lemnisci (5.00–9.32 mm long in the former *vs* about 10.0 mm in the latter) and different localities and hosts.

*Moniliformis moniliformis* is an important zoonotic acanthocephan species, parasitizing rodents, canines and felines worldwide, including China (Meyer, 1932; Petrochenko, 1958; Yamaguti, 1963; Ward and Nelson, 1967; Bhattacharya, 2007; Naidu, 2012). This species has a proboscis with 11–14 (usually 12) rows of 9–14 (usually 10–11) hooks each and much larger trunk (over 50.0 mm long in male), which is different from the new species (*vs* proboscis with 14 rows of 7–8 hooks each, and male 34.0–47.5 mm long in *M. tupaia* n. sp.). Additionally, molecular analysis displayed 27.3% and 17.2% nucleotide divergence in the *cox*1 and ITS regions, between the new species and *M. moniliformis*, which strongly indicated that they represent 2 distinct species. Dai *et al*. (2022) reported *Moniliformis* sp. XH-2020 from the plateau zokor (*Eospalax fontanierii baileyi*) in China, but they only provided the mitochondrial genomic data of their specimens (they did not describe the morphology). Pairwise comparison between *M. tupaia* n. sp. and *Moniliformis* sp. XH-2020 showed 27.3% and 23.6% nucleotide divergence in the *cox*1 and *cox*2 regions. Consequently, they belong to different species.

The class Archiacanthocephala currently includes 4 orders, namely Gigantorhynchida, Moniliformida, Oligacanthorhynchida and Apororhynchida (Amin, 2013). However, the phylogenetic relationships of the 4 orders remain unclear, due to a lack of genetic data of some taxa, especially the order Apororhynchida. The previous phylogenetic study using 18S or 18S + *cox*1 genetic data suggested a close affinity between Moniliformida and Gigantorhynchida (Amin *et al*., 2013, 2020). However, our phylogenetic results based on the 18S + *cox*1 data suggested Moniliformida is a sister to Oligacanthorhynchida, rather than Gigantorhynchida, which are consistent with some previous studies based on *cox*1 or 18S data (Gomes *et al*., 2020; Rodríguez *et al*., 2021; Amin *et al*., 2021, 2022). In the order Oligacanthorhynchida, the present phylogeny displayed *Nephridiacanthus major* nested in representatives of *Macracanthorhynchus* (clustered together with *M. ingens*), which challenged the current systematic position of this species. The present results agreed well with the previous phylogenetic study based on *cox*1 data (Rodríguez *et al*., 2021). In the genus *Moniliformis*, our molecular phylogenetic analyses indicate that *M. tupaia* n. sp. is a sister to *M. moniliformis*.

## Data Availability

The nuclear and mitochondrial DNA sequences of *M. tupaia* n. sp. obtained herein were deposited in the GenBank database [http://www.ncbi.nlm.nih.gov, accession numbers: PP002170, PP002171 (18S); PP002172, PP002173 (28S); PP002174, PP002175 (ITS); OR997666, OR997667 (*cox*1); PP002935, PP002936 (*cox*2)]. Type specimens of *M. tupaia* n. sp. (HBNU-A-M20231201-3CL) were deposited in the College of Life Sciences, Hebei Normal University, Hebei Province, China.
